# Commercial Local Pharmacotherapeutics and Adjunctive Agents for Nonsurgical Treatment of Periodontitis: A Contemporary Review of Clinical Efficacies and Challenges

**DOI:** 10.3390/antibiotics9010011

**Published:** 2019-12-30

**Authors:** Oi Leng Tan, Syarida Hasnur Safii, Masfueh Razali

**Affiliations:** 1 Faculty of Dentistry, Centre for Restorative Dentistry, Unit of Periodontology, National University of Malaysia, Jalan Raja Muda Abdul Aziz, Kuala Lumpur 50300, Malaysia; oileng1086@gmail.com; 2 Faculty of Dentistry, Department of Restorative Dentistry, University of Malaya, Kuala Lumpur 50603, Malaysia; syarida.safii@um.edu.my

**Keywords:** periodontitis, anti-infective agents, local, anti-bacterial agents, periodontal debridement, periodontal pocket

## Abstract

Periodontal infections tend to be site-specific, mostly confined to the periodontal pocket. With the surge of antibiotic-resistant bacteria, the trend is shifting towards other therapeutic modalities, especially locally delivered approaches that include other pharmacotherapeutic drugs and medical devices. This narrative review aimed to provide insights into the clinical efficacy of local drug delivery and adjunctive agents used in nonsurgical management of periodontitis. Electronic (PubMed/MEDLINE, CENTRAL, and EMBASE) and bibliographic searches of past systematic reviews were carried out to identify previous publications on the topic. Only relevant literature and randomized controlled trials published in English were selected. In addition, a literature review was developed based on the selected articles. Experimental drugs or agents were excluded. This review highlights the clinically proven and commercially available therapeutic agents related to the management of periodontal disease with comparisons of their clinical efficacies and challenges. A vast array of commercial local pharmacotherapeutic agents had been clinically tested, but the methodologies and clinical results varied within and between each agent used, causing difficulty in drawing conclusions and providing support to the superiority of one agent over another. Considering the benefit–cost ratio with the modest clinical results, the long-term usefulness of these agents remains debatable.

## 1. Introduction

Periodontitis is defined as an inflammatory disease of the periodontium with progressive destruction of tooth-supporting tissues. Accumulation of dental biofilm on the tooth surface [1] will trigger the imbalance between oral commensal microorganisms and host defense [2] in a susceptible individual, leading to the development of periodontitis under suitable conditions of bacterial environment and specific periodontopathogens. Several risk factors, such as tobacco smoking [3,4], host genetic variations [5,6], and certain systemic conditions [7,8], can also influence the progression and severity of periodontitis [9]. If left untreated, periodontitis may lead to tooth loss, causing substantial functional and aesthetic and psychological impact to the affected individuals [10].

Over 90% of the general world population are estimated to be suffering from a certain form of periodontal disease [11]. The recent Global Burden of Disease Study [12] ranked severe periodontitis as the 11th most prevalent disease, affecting 10.5% or 750 million people worldwide. Given that periodontitis involves microbial etiology and pathogenesis related to inflammation, pharmacologic approaches based on antimicrobials [13], probiotics [14,15], natural products [16,17], and host modulation [18] have garnered considerable research interest in the past three decades. The Acute Market Reports [19] revealed that the global periodontal therapeutics market, which was valued at US$259.5 million in 2016, would expand at a compound annual growth rate of 9.2% and is expected to reach US$580.5 million by the year 2025. With 30.4% of the global population smoking daily [20], combined with the rising diabetes epidemic [21], these established risk factors are likely to increase the periodontitis incidence and further drive the global periodontal therapeutics market.

Reviews on local drug delivery (LDD) systems are abundant. However, they focus on antimicrobials. With the advent of the new periodontal classification that had merged both chronic and aggressive periodontal disease entities together [22], this contemporary literature review aimed to provide an overview of the local drug delivery and adjunctive agents (LDA) used in periodontal treatment and highlight the clinically proven and commercially available therapeutic agents related to the management of periodontal disease with comparisons of their clinical efficacies and the challenges of their application. 

## 2. Classification of LDA for Nonsurgical Periodontal Therapy

**Figure 1:**
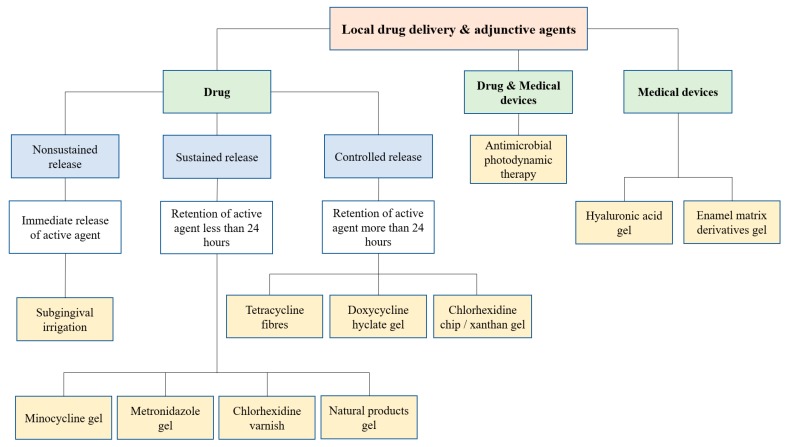
Classification of commercially available local drug delivery and adjunctive agents (LDA) for nonsurgical periodontal therapy.

LDD into periodontal pockets had been classically categorized as nonsustained-, sustained-, or controlled-release subgingival delivery [23,24]. With the introduction of adjunctive agents, including medical devices, we attempted to classify them, as illustrated in Figure 1. 

Nonsustained delivery commonly provides immediate release of the active agent by means of subgingival irrigation. Despite being categorized as LDD agents, mouthwash and supragingival irrigations cause no direct effect on subgingival microorganisms as they show no penetration of the gingival crevice or periodontal pockets [25]. Sustained-released devices are designed to carry high concentration of agents into periodontal pockets for a short duration (less than 24 h). By contrast, controlled delivery systems should be able to retain the active agent over an extended time period (more than 24 h) within the periodontal pockets [26].

A medical device is defined by the U.S. Food and Drug Administration (FDA) [27] as ‘an instrument, apparatus, implement, machine, contrivance, implant, in vitro reagent, or other similar or related articles, including a component part or accessory’ used in the diagnosis, treatment, or prevention of disease or other conditions or intended to affect the body’s structure or function not by the primary metabolism and chemical action within the body. To date, the adjunctive agents registered as medical devices under FDA include hyaluronic acid and enamel matrix derivatives (EMDs). In addition, antimicrobial photodynamic therapy contains both drug and medical device components. 

## 3. Indications for the Use of LDA in Periodontal Treatment

Enhanced patient compliance, improved efficacy, and few side effects have favored the use of local antimicrobials [28]. Evidently, LDD systems offer no advantages as a monotherapy [29]. Matesanz-Perez et al. [30] conducted a systematic review and meta-analyses on clinical studies evaluating the outcomes of locally delivered antimicrobials and found a statistically significant (*p* = 0.000) overall probing pocket depth (PPD) reductions and clinical attachment level (CAL) gains of 0.407 and 0.310 mm, respectively, when used as adjuncts to scaling/root planning (SRP) compared with mechanical debridement alone. 

Although one may find the resultant clinical parameters unremarkable, local antimicrobials have been advocated for local nonresponding or recurrent sites during supportive periodontal therapy [31], the presence of residual pockets in the aesthetic zone, in which surgery may compromise aesthetics or phonetics, and persistent bleeding pockets in the intrabony sites [32,33]. High-risk groups, such as smokers, diabetics, or those with erratic oral hygiene compliance and patients with relative or absolute contraindications to surgical intervention possibly benefit from the adjunctive effect of LDD [13,34,35].

## 4. Pharmacotherapeutic Agents Used as Local Adjuncts

**Figure 2:**
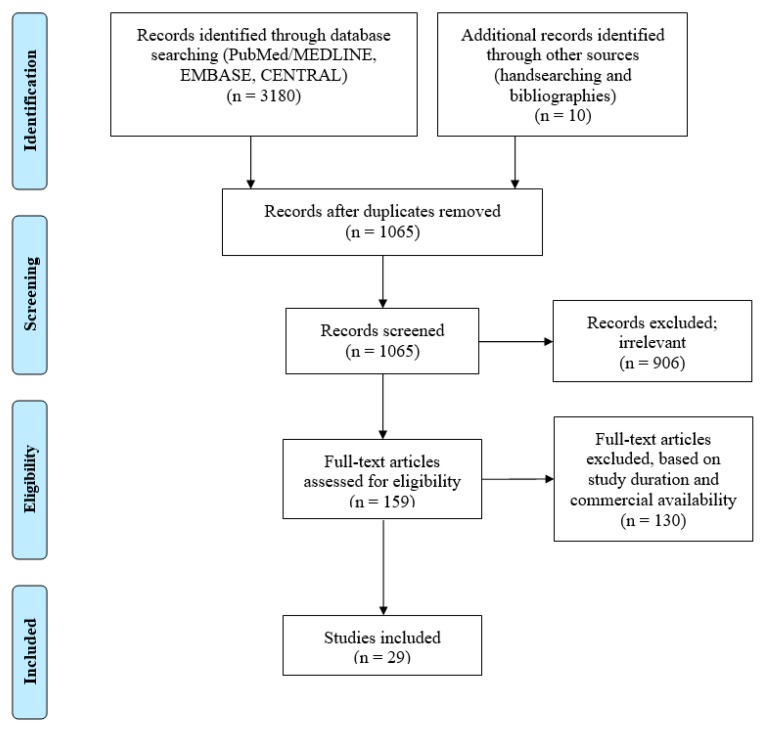
PRISMA (preferred reporting items for systematic reviews and meta-analyses) flow diagram.

A search was conducted using the PubMed/MEDLINE, CENTRAL, and EMBASE databases to identify any randomized controlled and professionally applied LDA used in healthy human intervention studies for treatment of periodontitis. The search considered works published from 1979 until November 2019 by using the keywords ‘periodont *’, ‘antimicrobial’, ‘photodynamic therapy’, ‘hyaluron *’, ‘enamel matrix derivative *’, ‘chlorhexidine’, ‘tetracycline’, ‘minocycline’, ‘metronidazole’, ‘doxycycline’, ‘non-surgical’, ‘scaling and root planing’, ‘adjunct’, ‘subgingival’, and ‘local delivery’. Bibliographies from previous systematic reviews on LDA were scrutinized [29,30,36,37,38]. Only relevant literature in English from electronic search were selected for the present review. The LDA had to be used as an adjunct and compared to a mechanical debridement control or placebo group. The use of systemic antimicrobials, banned, discontinued, and experimental drugs and agents were excluded. Search results were presented in accordance to Preferred Reporting Items for Systematic Reviews and Meta- Analyses (PRISMA) flow diagram (Figure 2). 

**Figure 3:**
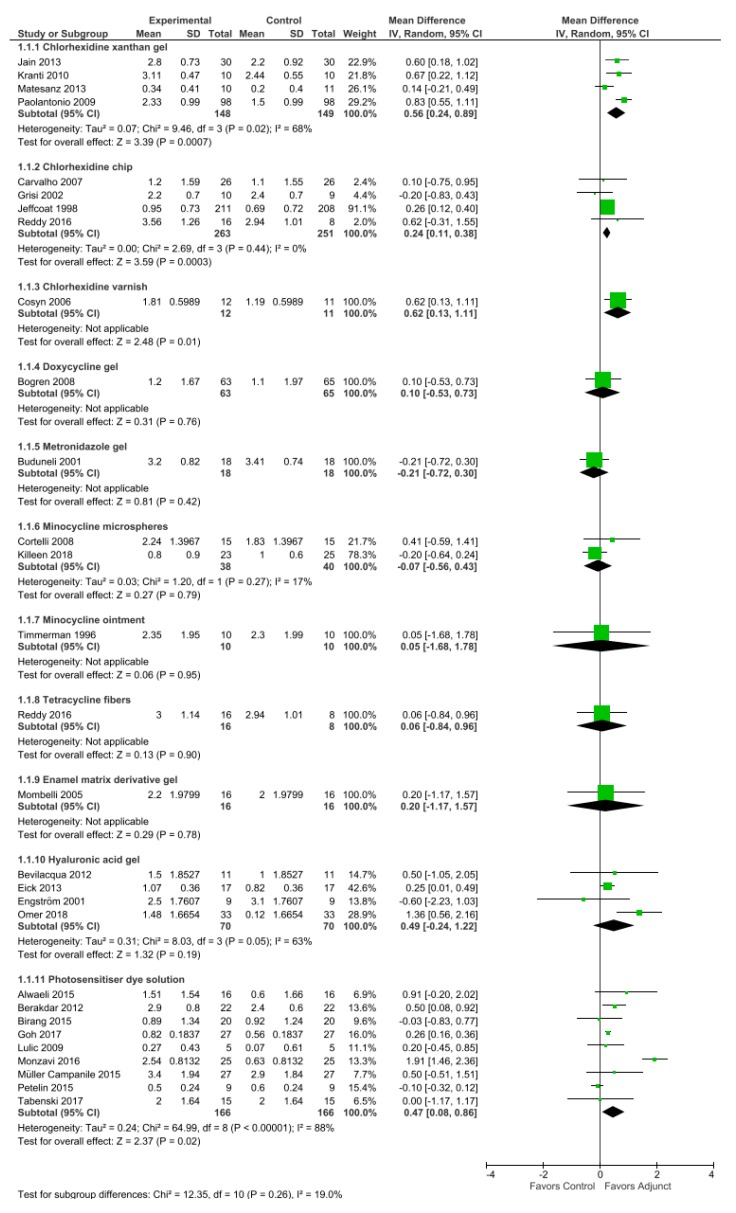
Meta-analysis of studies on changes in probing pocket depth (PPD) for control (mechanical debridement alone) versus use of adjuncts, sub-grouped by agent, based on random effects model; mean difference in units of millimeters.

**Figure 4:**
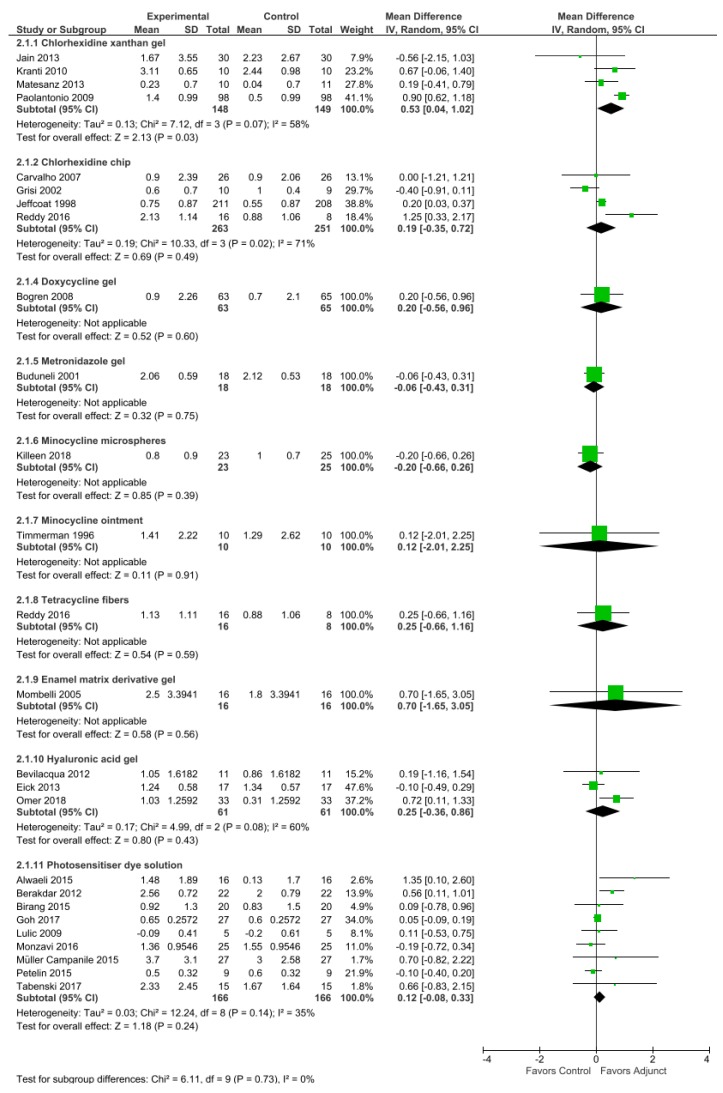
Meta-analysis of studies on changes in clinical attachment level (CAL) for control (mechanical debridement alone) versus use of adjuncts, sub-grouped by agent, based on random effects model; mean difference in units of millimeters.

**Figure 5:**
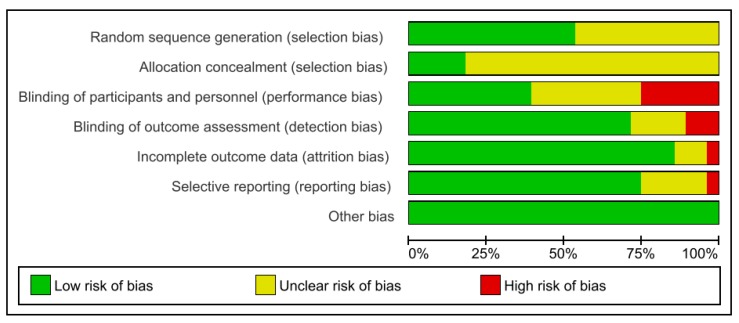
Risk of bias graph: Review authors’ judgements about each risk of bias item presented as percentages across all included studies.

Subsequent paragraphs include details on commercially available LDA reported in literature, with their clinical efficacies being based on the longest follow-up studies, as summarized in Table 1. Quantitative analysis was performed based on changes in PPD (Figure 3) and CAL (Figure 4) for the selected studies using RevMan 5.3 [39]. Risk of bias of included studies was presented as percentages in graph form (Figure 5). Six domains were assessed and judged as ‘low risk’, ‘unclear risk’, or ‘high risk’ of bias according to the Cochrane Handbook [40].

### 4.1. Tetracycline 

The first controlled-release LDD system to treat periodontitis, a device that included hollow fibers made of cellulose acetate containing tetracycline, was developed by Goodson and colleagues in 1979 [69]. With their bacteriostatic antimicrobial properties that inhibit bacterial protein synthesis, tetracyclines exhibit high substantivity to root surfaces [70] and periodontal pocket hard tissues [71,72]. However, a significantly long exposure time is required compared with other agents [73].

**Fibers:** Actisite^®^ tetracycline fiber (ALZA Corporation, Palo Alto, CA, USA) was the first commercially available controlled-released LDD introduced in 1994. This substance is nonresorbable but biologically inert and packaged as a 0.5 mm diameter ethylene and vinyl acetate copolymer imbued with 25% *w/w* tetracycline which is equivalent to 12.7 mg tetracycline hydrochloride. The active drug can be maintained at a constant concentration in the gingival crevicular fluid (GCF) in excess of 1300 µg/mL over a period of 10 days [71] compared with 8 µg/mL in systemic administration [74].

Despite its clinical efficacy, fiber insertion was found to be complicated and time consuming, requiring 7–10 min for application [75] and with specific transient gingival redness observed upon removal [76]. In a five-year controlled clinical trial, Wilson and co-workers [77] reported no significant differences between treatments compared with their preliminary six-month data, demonstrating that adjunctive tetracycline fiber therapy featured better clinical parameters than SRP alone. This finding suggests that combination therapy may only provide temporal initial advantages. Following the development of other new biodegradable agents, the fibers were subsequently discontinued in 2003 [78,79].

Periodontal Plus AB™ (Advanced Biotech Products, Chennai, India) is a bioresorbable tetracycline fiber developed based on a collagen film system. The substance is available in vials containing 25 mg fibrillar collagen impregnated with 2 mg tetracycline hydrochloride. Application of this fiber removes the need of a second appointment for fiber removal because it biodegrades within the pocket [80]. However, a 12-month study conducted using the product reported insignificant clinical benefits [48]. 

### 4.2. Doxycycline 

Doxycycline and minocycline are second-generation semisynthetic derivatives of tetracycline that exhibit superior antimicrobial activities compared with its predecessors, which include strains of tetracycline-resistant bacteria, owing to their improved binding properties and good absorption with prolonged duration of action [81].

**Gels:** Atridox^®^ (Atrix Laboratories, Fort Collins, CO, USA) is the first FDA-approved resorbable doxycycline gel system; it is composed of two syringes of powder and liquid that are mixed together with 25 times of repetition. With its capability to down-regulate matrix metalloproteinase [18], the 10% doxycycline hyclate thixotropic gel solidifies upon contact with the tissue fluid, and doxycycline levels can remain above 1000 µg/mL for 18 h in the GCF [82]. Studies have shown that doxycycline had persisted above the minimum inhibitory concentration (MIC) for periodontal pathogens (6.0 µg/mL) at local site for seven days, and 10–20 mg/mL of the detected drug was still observed at 3–5 days post-polymer removal [83]. A study conducted over a period of three years revealed improved clinical outcomes at three months; however, the findings failed to last until one year of follow-up examination, indicating that repeated annual application of doxycycline showed no long-term clinical and microbiological effect beyond mechanical debridement alone [50].

### 4.3. Minocycline

**Microsphere:** Minocycline in the form of microspheres, Arestin^®^ (OraPharma, Inc., Warminster, PA, USA) was FDA-approved in 2001. Each syringe contains 4 mg of 20–60 µm diameter bioresorbable microspheres, equivalent to 1 mg minocycline base in a poly (glycolide-lactide) carrier [84]. Initially in powder form, the polymer will hydrolyze immediately upon contact with GCF and adhere to the periodontal pocket. Administration will result in sustained release of minocycline concentration of 340 µg per mL through 14 days, exceeding the MICs for periodontopathogens [85]. Cortelli et al. [52] and Killeen et al. [53] failed to demonstrate that subgingival minocycline treatment enhances results of mechanical debridement in the long-term despite repeated application with three-month application in the former and six months in the latter over 24 months. 

**Ointment:** Dentomycin^®^ (Lederle Dental Division, Gosport, Hampshire, UK) and Periocline^®^ (Sunstar, Osaka, Japan) are both biodegradable minocycline 0.5 mg ointments consisting of 2% minocycline hydrochloride (10 mg minocycline) in a matrix of hydroxyethyl cellulose, aminoalkyl methacrylate, triacetine, and glycerine. In a controlled 18-month clinical trial involving subjects with moderate to severe chronic periodontitis, repeated intermittent subgingival application of the gel provided no benefits to the subjects [54]. The authors suggested that the negative results were due to their optimal oral hygiene instruction and reinforcement, wherein other protocols were not emphasized. 

### 4.4. Chlorhexidine

In addition to its immediate bactericidal action and prolonged bacteriostatic action on the tooth surface [86], chlorhexidine is a broad-spectrum antiseptic, which features a large dicationic molecule at physiological pH; this property enables chlorhexidine to bind to the bacterial cell wall and different surfaces within the mouth, with its substantivity maintaining antibacterial activity up to 12 h [87,88].

**Irrigation solution:** Various concentrations of chlorhexidine (0.02% to 0.2%) had been used in clinical studies. Two systematic reviews compared chlorhexidine subgingival irrigation as an adjunct to mechanical instrumentation, but both found no additional benefit to SRP alone [37,89]. The possible reasons for this finding could be the rapid clearance of the drug from the site due to constant GCF outflow [90] and/or the use of ineffective concentrations, which are further reduced in the pocket due to the high affinity of chlorhexidine for blood and salivary proteins [91,92,93].

**Chip:** A commercial FDA-approved biodegradable controlled-release chip containing 2.5 mg chlorhexidine gluconate in a gelatin matrix is sold under the trade name Periochip^®^ (Perio Products Ltd., Jerusalem, Israel). The small chip measuring 4.0 mm × 5.0 mm × 0.35 µm releases chlorhexidine in a biphasic manner, with an initial peak of 2007 µg/mL within 2 h in the GCF post-insertion followed by maintenance of high concentrations (above 1000 µg/mL) for the following 96 h and complete biodegradation between 7 and 10 days after insertion [94]. An alternative form called Periocol^®^-CG (Eucare Pharmaceuticals Pvt. Ltd., Chennai, India) incorporates 2.5 mg chlorhexidine into a collagen membrane chip derived from fresh water fish [95]. Currently, no study compares the two chips within the same clinical trial. The use of chlorhexidine chips resulted in improved clinical parameters in a nine-month study by Jeffcoat et al. [45] and in a 12-month study by Reddy et al. [48]. However, Carvalho et al. [47] and Grisi et al. [46] observed no clinical nor microbiological effect beyond conventional SRP over nine months. When grouped together, a weighted mean difference (WMD) of 0.24 mm in PPD reduction and 0.19 mm in CAL gain were observed.

**Gel:** Chlo-Site^®^ (Ghimas s.p.a., Casalecchio di Reno, Italy) is a xanthan-based chlorhexidine gel containing a combination of 0.5% chlorhexidine digluconate and 1.0% chlorhexidine dihydrochloride. The carrier comprises of a saccharide polymer, which can increase liquid viscosity, providing it with mucoadhesive property to stick to the pocket [96]. Although Jain et al. [43] reported that xanthan gel treatment group promoted good improvement of bleeding score and pocket depth reduction until six months after treatment, Matesanz et al. [44] found limited improvement in clinical outcomes with no significant difference between groups in their six-month study. A WMD of 0.56 mm in PPD reduction and 0.53 mm in CAL gain were seen from our review.

**Varnish:** Although widely used in caries prevention, chlorhexidine varnish also shows value in treating periodontal disease. A nine-month study by Cosyn et al. [49] reported a 0.62–1.06 mm reduction in pocket depth with application of EC40^®^ (Biodent BV, Nijmegen, The Netherlands), a highly concentrated solution of 35% chlorhexidine diacetate in 37% alcohol base stabilized by 27% sandarac, a naturally occurring resin. BioC^®^ (Biodent BV, Nijmegen, The Netherlands) is another variant of the varnish in supersaturated concentration of 20% chlorhexidine diacetate [97]. Both drugs are packaged in a syringe containing 1.5 mL agent. A different varnish, Cervitec^®^ Plus (Ivoclar/Vivadent AG, Schaan, Liechtenstein) comprising 1% chlorhexidine (equivalent to 10 mg/mL chlorhexidine) and 1% thymol in a viscous polyvinyl butyral base, was found to reduce anaerobic bacterial count within the periodontal pocket for up to three months with multiple applications [98,99].

### 4.5. Metronidazole 

Whenever antibiotics are considered for use in periodontal therapy, metronidazole has often been the drug of choice owing to its bactericidal activity against obligate anaerobes by inhibiting DNA synthesis [100].

**Gel:** Elyzol^®^ (Dumex, Copenhagen, Denmark) is a licensed drug that consists of 40% metronidazole benzoate in an oil-based (glyceryl mono-oleate and sesame oil) mixture which is slowly disintegrated by GCF enzymes into 25% metronidazole [101]. Upon subgingival application of the drug with a syringe applicator, it initially liquefies at body temperature and then changes to a highly viscous semisolid state upon contact with GCF [101]. According to Stoltze [102], metronidazole concentration above 1 µg/mL can be measured in the periodontal pocket up to 36 h, with concentrations above MIC_50_ for susceptible periodontopathogens 24 h after administration without any systemic side effects [103]. Buduneli et al. [51] showed an average reduction of 3.2 mm in PPD and mean CAL gain of 2.1 mm for scaling plus adjunctive metronidazole gel, but was not superior to that of conventional periodontal therapy.

### 4.6. Povidone–Iodine

Iodine utilization is well known in the world of medicine for its broad-spectrum bactericidal efficacy including periodontopathogens [104].

**Irrigation solution:** Sahrmann et al. [105] observed that the adjunctive use of povidone–iodine to SRP resulted in a minimally statistical additional benefit of 0.28 mm reduction in PPD with no reported adverse side effects. No correlation was observed between the clinical results and antiseptic concentrations, which had ranged from 0.1% to 10%, possibly because the antibacterial action of the drug increases with the dilution degree [106]. Thus, maximum bactericidal effect could still be achieved despite dilution of highly concentrated preparations by GCF and blood in the pocket. However, certain studies have reported no evidence of effectiveness with adjunct use of povidone–iodine subgingival irrigation [107,108,109].

### 4.7. Sodium Hypochlorite

Sodium hypochlorite is the most prominent chlorine used in dentistry especially in endodontic therapy [110]. The antiseptic agent, which is easily accessible and inexpensive, exhibits broad-spectrum antimicrobial activity that inhibits bacterial enzymes [111].

**Irrigation solution:** A study by Bizzarro et al. [112] reported that a single episode of 0.5% sodium hypochlorite subgingival irrigation showed no significant clinical and microbiological improvement compared with mechanical therapy alone. 

### 4.8. Natural Products

**Gel:** NBF Gingival Gel (NanoCureTech Co., Ltd., Seoul, South Korea) is a nanoemulsion gel with ingredients of vitamin C, vitamin E, propolis, aloe, and green tea extract, with claims of being an antibacterial, anti-inflammatory, and anti-oxidative. Debnath et al. [113] reported a statistically significant improvement in periodontal PPD and CAL at three months post-application compared with mechanical therapy alone with a difference of 0.74 and 0.71 mm, respectively, between both groups. However, it was the only study that utilized the gel as adjunct for periodontitis treatment with six participants therefore its efficacy is debatable.

### 4.9. EMD

Enamel matrix proteins were demonstrated in clinical studies as being secreted from Hertwig’s epithelial root sheath; therefore, they can promote new periodontal attachment formation [114,115,116]. Extracted from purified porcine embryonal enamel, the proteins were renamed as EMDs, and they have been broadly used in periodontal regenerative surgery to treat intrabony, furcation, and recession defects [117]. EMD is assumed to mimic the role of enamel matrix proteins in cementogenesis by inducing new cementum formation and stimulating matrix deposition on native cementum [118,119].

**Gel:** Emdogain^®^ (Institute Straumann AG, Basel, Switzerland) is a gel containing 30 mg/mL EMD in a propylene glycol alginate carrier, which is available as 0.15, 0.3, and 0.7 mL syringes. The amelogenin content precipitates when in contact with physiological pH and body temperature, forming an insoluble protein layer on the root surface that remains for up to four weeks [120]. Three randomized controlled clinical studies compared the adjunctive use of EMD with the nonsurgical periodontal therapy [55,121,122]. Although the previous two studies reported no significant additional benefits with EMD, Graziani et al. [122] observed that application in deep pockets (≥6 mm) resulted in low D-dimer levels, indicating lower fibrinolysis and better periodontal healing of periodontal pockets compared with mechanical debridement alone. However, more studies would be needed to confirm its efficacy as an adjunct in the nonsurgical treatment of periodontitis for only one study had a long-term follow-up [55].

### 4.10. Hyaluronic Acid

Hyaluronan, a high-molecular-weight glycosaminoglycan, is an important constituent of the extracellular matrix of mineralized and nonmineralized tissues and particularly abundant in the nonmineralized component of periodontium [123,124]. In periodontal disease, the high-molecular-weight hyaluronan synthesized in the periodontal tissue undergoes massive degradation, transforming into its low molecular form as a result of bacterial enzyme action (hyaluronases) [125,126,127]. Thus, topical application of hyaluronan to inflamed periodontal sites was deemed to possess potential in inducing periodontal healing due to its role as a key element in wound repair [128,129].

**Gel:** Several commercial brands have been clinically tested for use in periodontal disease; these brands include Healon GV^®^ (Pharmacia and Upjohn, Uppsala, Sweden), which is available in 0.85 mL syringes containing 14 mg/mL sodium hyaluronate derived from rooster’s comb [59]; Aminogam^®^ (Errekappa Euroterapici, Spa, Italy) contains non-animal origin sodium hyaluronate combined with a pool of synthetic amino acids [57]; Aftamed^®^ (BioPlax Limited, London, UK) includes 240 mg/100 g synthetic sodium hyaluronate [56]; Gengigel^®^ (Ricerfarma S.r.l., Milano, Italy), which contains sodium hyaluronate is derived from bacterial fermentation (*Streptococcus equi*.) at concentrations of 0.2% [130] and 0.8% [58,131]. Their mucoadhesive properties are derived from polyacrylic acid crosslinked with a divinyl glycol (polycarbophil) carrier. WMD of 0.49 mm PPD reduction and 0.25 mm CAL gain were demonstrated based on four studies [56,57,58,59].

### 4.11. Antimicrobial Photodynamic Therapy (aPDT)

aPDT is an emerging trend in dentistry. This procedure utilizes a locally applied photosensitizer that is absorbed by bacteria, and upon irradiation with a specific wavelength light source, the photosensitizer would be activated and generate a cytotoxic singlet and triplet oxygen that disintegrates bacterial membranes [132,133]. aPDT applications have been shown to reduce microbial load [134,135], and studies have revealed that aPDT can be beneficial and relatively safe to adjunctively treat periodontitis [136,137]; however, whether aPDT could replace the use of antimicrobials remains inconclusive [138,139].

**Dye solution:** Several different types of photosensitizers, including methylene blue [66,67], toluidine blue [68], phenothiazine chloride [62,63,64,65], and indocyanine green [60,61], were studied for periodontal therapy use. All these dyes possess bactericidal properties and differ in their activation wavelength; therefore, the choice of photosensitizers is dependent on the light source used [140,141]. Commercial diode laser systems marketed for aPDT adjunctive use in periodontal therapy usually carry their own compatible photosensitizer dyes. Four commercially available aPDT systems demonstrated a WMD of 0.47 mm PPD reduction and 0.12 mm CAL gain in our review.

## 5. Occurrence of Adverse Effects with Use of LDA

While there are studies that observed no adverse events, safety and adverse effects with the use of LDA were often not reported in most of the studies. The few studies that described them included feeling of illumination of the eye with the use of laser [67], gingival tenderness after application of gel [54], experience of pain following therapy [49], dislodgment of chip after placement [47], gingival edema or abscesses [46], fever, headache, toothache, discomfort, and sensitivity [45].

## 6. Comparison of Clinical Efficacy between Different LDA

As documented, mechanical therapy alone is an effective treatment with long-term success in majority of affected individuals [142]. Although some individuals might respond inadequately to treatment, adjuncts may be pivotal in such cases, especially if surgical options are inapplicable. In general, all systematic reviews and meta-analyses that previously evaluated the efficacy of LDA systems had shown minimal but positive clinical results as adjuncts compared with mechanical therapy alone (summarized in Table 2). 

However, not all adjuncts showed additional benefits. Hanes and Purvis [37] reported no adjunctive advantage from the application of local subgingival antiseptic irrigants during or immediately after mechanical debridement, which is probably due to the rapid clearance by GCF flow [90]. Subsequent reviews excluded subgingival irrigation as a comparator; however, recent studies are still investigating their efficacy [112,143,144]. Thus, future meta-analyses should include these studies for clinicians to contemplate on their usage in their daily practice.

Most previous studies compared a single form of LDD against mechanical therapy rather than between other systems, and only a handful included more than one test group [145,146,147,148]. Many of these studies, including the recent ones, involved a short follow-up period of three months and less. Table 1 lists the longest duration of randomized clinical trials conducted for each LDA. 

Smiley et al. [29] conducted the most comprehensive review to date on efficacy of adjuncts, with a recent supplement network analysis [36] determining that doxycycline hyclate gel and photodynamic therapy with diode laser featuring the greatest likelihood for CAL gain and with the results being retained until six months and beyond. With the aim of providing a companion evidence-based piece for the American Dental Association Clinical Practice Guidelines, the authors only included adjuncts available in the United States of America, therefore limiting the external validity of the review. Thus, the question of which LDA system is superior remains unanswered. 

The authors had included both systemic and local adjuncts in their review. Comparing local and systemic antimicrobials might be inappropriate given that they feature their own indications and merits; however, the adjunctive benefits reported nearly similar clinical outcomes comparable with locally delivered antimicrobials, ranging from 0.2 mm to 0.8 mm PPD reduction and CAL gain when used to treat chronic periodontitis [29,149]. Several notable benefits of systemic antimicrobials use were observed in aggressive cases, with CAL gain of up to 1.0 mm after 12 months [149].

## 7. Use of LDA in Nonsurgical Treatment of Aggressive Periodontitis

The recent 2017 World Workshop on the Classification of Periodontal and Peri-implant Diseases and Conditions had removed the term ‘aggressive periodontitis’ as previously defined by the 1999 Classification Workshop [150], citing insufficient evidence to distinguish both chronic and aggressive forms as separate diseases having distinct etiology and pathophysiological elements [22]. Literature on LDA that has been mentioned in this paper so far had focused on adjuncts used in the management of chronic periodontitis. Although there is no consensus on the use of antibiotics in managing periodontitis, adjunctive systemic antibiotics had been shown to have more pronounced clinical outcomes in aggressive forms of the disease [13,151]. Therefore, it would be expected that reviews on the adjunctive use of LDA in nonsurgical treatment of aggressive periodontitis would be lacking. Currently, there are three systematic reviews on the application of aPDT published in the same year, however with conflicting results on its beneficial use [152,153,154].

## 8. Challenges of LDA

Despite their indications for use, certain local delivery commercial devices had been discontinued due to the lack of profits, registration difficulties, and/or the costly requirements for continued approval [151]. The issue of substantivity [155] and rapid replacement of GCF within an inflamed periodontal pocket [90] posed a challenge for local mode of agent delivery. Substantivity by binding to soft or hard tissues surfaces is crucial to establish a reservoir for the drug to be slowly released within the pocket [156]. Goodson [90] estimated that GCF is replaced approximately 50 times per hour in a moderate periodontal pocket (4–5 mm).

An ecological concept describes periodontal disease as a mouth infection involving the tongue, saliva, and oral mucosa [157,158,159], limiting the use of localized agent therapy to residual deep periodontal pockets in the maintenance phase [160,161]. Clinicians should be aware that application of local adjuncts should not be used to overcome the shortcomings of adequate subgingival debridement. Bearing in mind the global rise of antimicrobial resistance, the usage of antibiotics should only be limited for clear indications [162]. Concerns indicate that the high concentrations used in local delivery may suppress or eliminate normal microbiota and initiate the development of antibiotic-resistant species within the pocket itself [163]. However, studies have proven that such concerns were unfounded as no cases [164,165] or only a transient increase in resistance bacteria with no permanent change to the microbiota was observed [166].

## 9. Cost Effectiveness and Patient-Centered Outcomes

An optimum periodontal condition obtained through periodontal therapy is important to a clinician; in addition, decreasing the need for surgical interventions during the process is not only valuable economically, but also for patient-centered outcomes [167,168,169]. A systematic review by Niederman et al. [170] analyzed the cost effectiveness of local antimicrobial drugs that included the cost of agent, consumption, quantity of drug placements, mechanical therapy, and the working time of the clinician. The authors concluded that treating a single tooth at a dedicated visit would be the most expensive treatment (US$99 to US$126 per tooth in 2002) compared with treatment of a quadrant (US$28 to US$46 per tooth in 2002). Moreover, treating a quadrant as an added procedure would cost as low as US$20 (2002) per tooth as the setup cost is covered by the first procedure. Thus, using adjuncts for periodontal maintenance in an economic sense is favorable.

Considering treatment time, Wennström et al. [171] compared SRP with debridement (ultrasonic instrumentation) combined with LDD and observed that the latter procedure required less total treatment time (2 h against 3 h 11 min) in six months periodontal therapy. Adverse events were relatively minor with the use of LDD systems [37,38]. Meanwhile, Braegger [172] reported that local delivery devices feature potential cost savings and are less time consuming compared with conventional mechanical treatment given their similar six-month clinical outcomes. Heasman et al. [173] argued that cost effectiveness of systemic antimicrobials are pronounced through economic analysis. However, the analysis failed to consider that patient management could be influenced by the risk of increased bacterial resistance.

## 10. Limitations

There are plenty of studies published on LDA used in the nonsurgical treatment of periodontitis. However, the methodologies and clinical results varied within and between each agent used, causing difficulty in drawing conclusions and providing support to the superiority of one agent over another. Moreover, there is a lack of uniformity in the definition of periodontal disease severity. With the introduction of the new periodontal disease classification, we hope researchers would implement them in their future research. Our current review is limited by our selection of articles published only in the English language and use of commercial agents dictated by the length of their studies. This may lead to bias in the results and interpretations. Quantitative analysis was not performed for certain LDA as a minimum of two studies would be required [174].

## 11. Conclusions

LDA may still play a role in the management of periodontal disease, especially in combination with nonsurgical periodontal therapy, and could provide benefits in certain clinical situations as aforementioned. Overall, a vast array of commercially available local pharmacotherapeutic agents had been clinically tested. Based on our review of adjuncts with the longest follow-up studies, the mean differences of PPD reduction ranged from −0.21 to 1.91 mm and −0.56 to 1.35 mm of mean CAL gain. In general, most of these adjunctive agents had shown minimal but positive clinical results compared with mechanical debridement alone. However, considering the benefit–cost ratio with the modest clinical results, their long-term usefulness remains debatable. Additional carefully designed medium- to long-term randomized controlled studies preferably with a minimum six-month duration and stringent methodological criteria will be required for an accurate, universal assessment of the efficacy and sustainability of LDA before any ‘gold standard’ local adjunct could be recommended. 

## Figures and Tables

**Table 1:** Summary of clinically tested commercial LDA for nonsurgical periodontal therapy based on longest follow-up period.

Active Agent	Brand	Manufacturer	Dosage	Delivery Vehicle	Application and Duration (per Manufacturer/Study Design)	Longest Follow-Up Study	Authors	Study Design	Sample Size
**Chlorhexidine**	Chlo-Site^®^	Ghimas Company, Italy	1.5% CHX	Gel	1 application 15 days treatment	6 months	Paolantonio et al. 2009 [41]	Split-mouth	98
							Kranti et al. 2010 [42]	Split-mouth	10
							Jain et al. 2013 [43]	Split-mouth	30
							Matesanz et al. 2013 [44]	Parallel	22
	Periochip^®^	Perio Products Ltd., Jerusalem, Israel	2.5 mg CHX gluconate	Chip	1 application 7 days treatment	9 months	Jeffcoat et al. 1998 [45]	Parallel *	419
							Grisi et al. 2002 [46]	Parallel *	20
							Carvalho et al. 2007 [47]	Split-mouth *	28
	PerioCol^®^-CG	Eucare Pharmaceuticals Ltd., Chennai, India	2.5 mg CHX gluconate	Chip	1 application 7 days treatment	12 months	Reddy et al. 2016 [48]	Parallel	48
	EC40^®^	Biodent BV, Nijmegen, The Netherlands	35% CHX diacetate	Varnish	1 application 7 days treatment	9 months	Cosyn et al. 2006 [49]	Parallel	26
**Doxycycline**	Atridox^®^	Atrix Laboratories, Fort Collins, CO, USA	10% DOXY hyclate	Gel	1 application 7 days treatment	36 months	Bogren et al. 2008 [50]	Parallel *	132
**Metronidazole**	Elyzol^®^	Dumex, Copenhagen, Denmark	25% MET benzoate	Gel	2 applications 7 days treatment	12 months	Buduneli et al. 2001 [51]	Split-mouth	18
**Minocycline**	Arestin^®^	OraPharma, Inc., Warminster, PA, USA	1 mg MINO hydrochloride	Micro-spheres	1 application 14 days treatment	24 months	Cortelli et al. 2008 [52]	Parallel *	30
							Killeen et al. 2018 [53]	Parallel *	55
	Dentomycin^®^	Lederle Dental Division, Gosport, Hampshire, UK	2% MINO hydrochloride	Ointment	3–4 applications 14 days treatment	18 months	Timmerman et al. 1996 [54]	Parallel *	20
	Periocline^®^	Sunstar Corp., Tokyo, Japan							
**Tetracycline**	Periodontal Plus AB^TM^	Advanced Biotech Products, Chennai, India	2 mg TET hydrochloride		1 application 7 days treatment	12 months	Reddy et al. 2016 [48]	Parallel	48
**Enamel matrix derivative**	Emdogain^®^	Institute Straumann AG, Basel, Switzerland	30 mg/mL porcine enamel matrix derivative	Gel	1 application	12 months	Mombelli et al. 2005 [55]	Split-mouth	16
**Hyaluronic acid**	Aftamed^®^	BioPlax Limited, London, UK	240 mg/100 g sodium hyaluronate	Gel	1 application	6 weeks	Omer at el. 2018 [56]	Split-mouth	33
	Aminogam^®^	Errekappa Euroterapici, Spa, Italy	Sodium hyaluronate, amino acids		1 application	3 months	Bevilacqua et al. 2012 [57]	Split-mouth *	11
	Gengigel^®^	Ricerfarma, Italy	0.2% and 0.8% Sodium hyaluronate		1 application	6 months	Eick et al. 2013 [58]	Parallel *	42
	Healon GV^®^	Pharmacia and Upjohn, Uppsala, Sweden.	14 mg/mL sodium hyaluronate		3 applications 27 days treatment	12 months	Engstrüm et al. 2001 [59]	Split-mouth *	9
**Photosensitiser**	EmunDo^®^	A.R.C. laser GmbH, Germany	Indocyanine green (iodide-free)	Dye Solution	2–4 applications 14–27 days treatment	3 months	Birang et al. 2015 [60]	Split-mouth	20
							Monzavi et al. 2016 [61]	Split-mouth *	25
	HELBO^®^	Bredent Medical, Germany	Phenothiazine chloride		1 application	12 months	Lulic et al. 2009 [62]	Parallel	10
							Alwaeli et al. 2015 [63]	Split-mouth *	21
							Petelin et al. 2015 [64]	Parallel *	27
							Tabenski et al. 2017 [65]	Parallel	48
	Periowave^TM^	Periowave Dental Technologies Inc, Canada	Methylene blue		1–3 applications	6 months	Berakdar et al. 2012 [66]	Split-mouth	22
							Müller Campanile et al. 2015 [67]	Split-mouth *	28
	Fotosan^®^	CMS Dental, Copenhagen, Denmark	Toluidine blue/tolonium chloride		1–3 applications	6 months	Goh et al. 2017 [68]	Split-mouth	27

CHX: Chlorhexidine; MINO: Minocycline; DOXY: Doxycycline; MET: Metronidazole; TET: Tetracycline. * Repeated application.

**Table 2:** Summary table of systematic reviews with meta-analyses comparing clinical efficacy of different LDA for nonsurgical periodontal therapy (scaling/root planning (SRP)).

Author and Year (Ref.)	Study Period	Types of Studies	Treatment Arms	Weighted Mean Differences (WMD) (mm) [95% Confidence Interval (CI)]		Main Outcomes and Conclusion
				Probing Pocket Depth (PPD)	Clinical Attachment Level (CAL) *	
**Hanes and Purvis [37]**	≥3 months	28 RCT, 2 CCT, 2 cohort	1. CHX, 2.5 mg in gelatin matrix	0.35 [n/a]	0.16 [n/a]	SRP alone showed sample-size adjusted mean reduction in PD of 1.45 mm (*p* = 0.002; CI = 0.56, 2.34), and adjusted mean gain in CAL was 0.89 mm (*p* = 0.001; CI = 0.55, 1.24). Adjuncts WMD for PD reduction ranged from 0.06 mm to 0.51 mm. WMD for CAL ranged from −0.40 mm to 0.39 mm Significant PD reduction was reported for MINO gel and microencapsulated MINO. Significant CAL gain was observed in studies of CHX chip and DOXY gel. All local CHX irrigation studies compared with SRP alone showed no additional benefits. Adverse events were reported to be infrequent and minimal, with local effects of instrumentation and/or drug application contributing to majority of it.
			2. MINO, 2% gel or ointment; microencapsulated powder	0.36; 0.26 (micro) [n/a]	0.39; −0.40 (micro) [n/a]	
			3. DOXY, 8.5% in biodegradable matrix; 15%	0.51 [n/a]	0.34 [n/a]	
			4. MET, 5%; 25% gel	0.06 [n/a]	0.07 [n/a]	
			5. TET, 25% fiber	0.21 [n/a]	−0.17 [n/a]	
			6. Sanguinarine, 5% gel	n/a	n/a	
			7. CHX, 2%, 12%, and 0.2% irrigation; ethyl cellulose	n/a	n/a	
**Bonito et al. [38]**	No minimum duration	50 RCT	1. TET	0.47 [0.22, 0.72]	0.24 [0.07, 0.42]	Adjunctive local antibiotics had PD reductions in the range of approximately 0.25 mm to 0.50 mm, and CAL gains in the range of approximately 0.10 mm to 0.50 mm. The most promising adjunctive therapy by combining PD and CAL results were suggested to be local MINO, followed by local tetracycline. Adverse events reported from these adjunctive therapies are relatively minor. Whether the improvements are clinically meaningful is still doubtful.
			2. MINO	0.49 [0.40, 0.58]	0.46 [0.32, 0.60]	
			3. MET	0.32 [0.20, 0.44]	0.12 [0.01, 0.24]	
			4. CHX	0.24 [0.13, 0.35]	0.16 [0.04, 0.28]	
			5. Other antibiotics (DOXY; ofloxacin)	n/a	n/a	
			6. Other antimicrobials (amine fluoride; stannous fluoride; triclosan; hydrogen peroxide; povidone iodine; tetra-potassium peroxy-diphosphate)	n/a	n/a	
**Matesanz-Pérez et al. [30]**	No minimum duration	52 RCT	1. CHX chip	0.328 [0.447, 0.209]	0.218 [0.329, 0.107]	Statistically significant (*p* = 0.000) overall results was observed for both changes in PD (WMD 0.407 mm) and CAL (WMD 0.310 mm) No significant differences were observed for bleeding on probing and plaque index. Substantial benefit in PD reduction (WMD between 0.5 and 0.7 mm) demonstrated with subgingival application of tetracycline fibers, sustained released DOXY and MINO. Minimal effect was observed with the local application of CHX and MET when compared with placebo (WMD between 0.1 and 0.4 mm).
			2. CHX varnish	0.413 [0.655, 0.170]	0.029 [0.550, −0.492]	
			3. CHX xanthan gel	n/a	0.891 [0.914, 0.867]	
			4. DOXY	0.573 [0.778, 0.367]	0.218 [0.260, 0.176]	
			5. MET	0.157 [0.303, 0.011]	−0.008 [0.091, −0.107]	
			6. MINO	0.472 [0.520, 0.424]	0.189 [0.251, 0.126]	
			7. TET fiber	0.727 [0.759, 0.695]	0.327 [0.552, 0.101]	
			8. TET strip	n/a	0.463 [0.401, 0.163]	
**Smiley et al. [29]**	≥6 months	72 RCT	1. SDD	n/a	0.35 [0.15, 0.56]	SRP alone had approximately 0.5 mm average improvement in CAL. A range of average CAL improvements between 0.2 and 0.6 mm was demonstrated in the combinations of assorted adjuncts compared with SRP alone. Moderate level of certainty for benefits in four adjunctive therapies compared with SRP alone: SDD, systemic antimicrobials, CHX chips and photodynamic therapy with a diode laser. Low level of certainty for benefits of the other included adjunctive therapies.
			2. Systemic antimicrobials		0.35 [0.20, 0.51]	
			3. CHX chips		0.40 [0.24, 0.56]	
			4. DOXY hyclate gel		0.64 [0.00, 1.28]	
			5. MINO microspheres		0.24 [−0.06, 0.55]	
			6. PDT with diode laser		0.53 [0.06, 1.00]	
			7. Diode laser		0.21 [−0.23, 0.64]	
			8. Nd:YAG lasers		0.41 [−0.12, 0.94]	
			9. Erbium lasers		0.18 [−0.63, 0.98]	
**John et al. [36] ^#^**		61 RCT		n/a	n/a	Network meta-analysis identified DOXY hyclate and photodynamic therapy with diode laser as having the highest probabilities for ranking first and second SRP adjuncts in terms of CAL gain, respectively. Adjuncts to SRP improved the response to SRP by 0.32 mm CAL over 6–12 months with no significant differences among the groups. Evident publication bias was observed, and the lack of studies inflated the treatment effects by an estimated 20%.

RCT: Randomized controlled trial; CCT: Case-controlled trial; n/a: not available; CHX: Chlorhexidine; MINO: Minocycline; DOXY: Doxycycline; MET: Metronidazole; TET: Tetracycline; SDD: sub-antimicrobial-dose doxycycline; Nd:YAG: Neodymium:yttrium-aluminum-garnet. * Negative (minus) sign indicates mean loss in CAL. ^#^ Network analysis of systematic review by Smiley et al. [29].
